# Tuberculosis in Patients with Haematological Malignancies

**DOI:** 10.4084/MJHID.2014.026

**Published:** 2014-04-07

**Authors:** Luis Anibarro, Alberto Pena

**Affiliations:** 1 Unidad de tuberculosis. Servicio de Medicina Interna. Complexo Hospitalario de Pontevedra, 36001-Pontevedra, Galicia, Spain; 2 Instituto de Investigación Biomédica de Vigo (IBIV). Vigo, Spain.

## Abstract

Tuberculosis (TB) is an infectious disease that causes more than 1 million deaths worldwide every year. In addition, it is estimated that one third of the world population is infected with M. tuberculosis in a latent state, which involves an eventual risk of progressing to active TB disease. Patients with immunodeficiencies, such as those suffering from haematological malignancies, have a greater risk of progressing to TB disease once infected. It is estimated that the Relative Risk of TB disease in patients with hematologic malignancies is 2–40 times that of the general population. The diagnosis of TB in these patients is often challenging as they often present clinical characteristics that are distinct to those of patients without any other underlying disease. Mortality due to TB is higher. Therefore, it is recommended to diagnose latent TB infection and consider preventive therapy that could avoid the progression from a latent state to active TB disease. There are currently two methods for diagnosing latent TB infection: the Tuberculin Skin Test (TST) and the Interferon-Gamma Release Assays (IGRA). Due to the lack of sensitivity in patients with immunodeficient conditions, a combined TST-IGRA testing is probably the best way for latent TB diagnosis in order to gain sensitivity. Treatment of latent TB infection and TB disease should follow the general principles to that in the general population.

## Introduction

Tuberculosis (TB) ranks as one of the leading causative diseases of mortality worldwide, with 8.6 million of new cases in 2012 and 1.3 million deaths attributable to the disease.^1^ Moreover, it is estimated that one third of the world’s population is infected by *M. tuberculosis*, which involves an eventual risk of progressing to active TB. Patients with immunosuppression, either due to underlying disease or of drug origin, are especially susceptible to developing the disease.^2^ In this article we perform a general overview of the clinical and epidemiological characteristics of TB in patients with haematological malignancies (HM), as well as its diagnosis and treatment.

## Pathogenesis of Tuberculosis

*M. tuberculosis* is the causative agent of TB. It is an acid-fast bacillus, weakly gram-positive and belongs to the genus *Mycobacterium* that includes more than 50 species.^3^ *M. tuberculosis* is a member of the *M. tuberculosis complex.* Only a few members of the *M. tuberculosis complex* are able to cause the disease in humans: *M. tuberculosis, M. bovis, M. africanum, M. canetii, and M. caprae*, being *M. tuberculosis* the responsible for the vast majority of TB cases in humans.^4^–^7^ As with the other species of the genus *Mycobacterium*, *M. tuberculosis* has a cell wall that is very rich in lipids (up to 40%), which makes it difficult to stain because of its acid-alcohol resistance. This characteristic has been the historical basis for its microbiological diagnosis by selective staining. Its rate of growth is very slow, even in specific media. Its generation time is around 18 hours, which is extremely long when compared to other species with a high prevalence of infection, such as *S. pneumoniae* or *E. coli* (20–30 minutes).^8^ Together with its ability to evade the human immune response, it is precisely the characteristic slow replication of *M. tuberculosis* that makes this species so difficult to eliminate and is probably the best explanation for its high historical prevalence.

Infection by *M. tuberculosis* starts with inhalation of droplets of 1–5 μm in diameter (Flügge droplets) that contain tubercle bacilli from a person with pulmonary TB.^9^,^10^ There are also other less usual alternative routes of transmission, such as the digestive tract, typical of *M. bovis,* through the ingestion of contaminated milk from infected livestock lacking proper veterinary disease control.

After reaching the alveolar space one of the following three scenarios can occur:

- Direct elimination of the bacillus.^11^
- Latent tuberculosis infection (LTBI) in which the tubercle bacillus resists the bactericidal mechanisms of alveolar macrophages, although without causing the disease.^12^,^13^ In this situation, the immune response is able to contain the bacillus, but not to eliminate it.

When the immune response is not able to contain *M. tuberculosis*, there is a development of TB disease, resulting in active TB. This situation can occur some months after initial infection (primary TB) or after reactivation of a prior LTBI (post-primary TB).

Overall, around 10% of people infected will go on to develop active TB,^2^,^14^ half of these within the first two years of exposure to the bacillus.^15^

The major immune response to infection by *M. tuberculosis* is focused on the type 1 helper T lymphocytes (Th1) capable of synthesizing interferon-gamma (IFN-γ) and other cytokines, which manage to contain *M. tuberculosis* in a latent state without active replication. The final situation between LTBI and active TB will depend upon the balance established between the immune response and the bacteria. According to the predominance of one or the other, either a potentially persistent latent state will be established or active TB disease will be generated. However, the concept of latent state-disease should not be understood as exclusively unidirectional compartments. There is a dynamic balance than can be bidirectional, and even includes “frontier” situations between both states, for which terms like “subclinical TB” or “incipient TB” have been proposed.^16^–^18^

## Risk of Developing Tuberculosis in Patients with Haematological Malignancies

As mentioned above, although the “frontier” between LTBI and active TB is not always clear or well defined, there is a series of individual and epidemiological situations that favour the acquisition of infection and/or development of the disease, such as HIV infection, recent exposure to a contagious TB patient, silicosis or immunodeficient conditions.^2^

Despite the heterogeneity of the immunological substrate presented depending on the specific type of malignancy, patients with HM have an underlying immunological deficiency that facilitates the emergence of infections.^19^,^20^ Alteration in the Th1 cell response of the HM itself or that caused by antineoplastic chemotherapy or hematopoietic stem cell transplantation (frequently associated to the administration of high doses of corticosteroids) lead to an impaired immune response that particularly promotes the progression from LTBI to active TB.^21^,^22^

For more than forty years, it has been known in clinical practice that patients with malignancies, especially those with HM, have a higher risk of developing TB than the general population.^21^,^23^–^28^ Table 1 gives details of some of the most significant studies that have analysed the risk of patients developing TB (search strategy in Pubmed with keywords: (“tuberculosis” or “latent tuberculosis infection”) and (“hematologic neoplasms” or “lymphoma”, “leukemia”, “multiple myeloma”, “stem cell transplantation”), as well as relevant references obtained from the selected articles or reviews papers on tuberculosis and cancer).^21^,^26^,^27^,^29^–^32^

The risk of developing TB can vary depending on the type of HM. Some authors have analysed this aspect, but the results of the studies differ. In a study undertaken in Brazil of more than 900 patients with HM, Silva FA et al found that Chronic Lymphocytic Leukemia (CLL) was the disease that showed a greater susceptibility to developing TB. In this same study, treatment with corticoids and fludarabine was also associated to a higher risk of developing the disease.^33^ However, in a retrospective study of around 3000 onco-hematological patients, Chen CY et al found that patients with Acute Myeloid Leukemia had a significantly higher incidence of TB disease than other subtypes of HM (2.87% vs. 1.21%, p=0.002).^32^

## Diagnosis of Latent Tuberculosis Infection

The aim of diagnosing LTBI in patients with HM is the early detection of infection by *M. tuberculosis* while still in the latent phase, so treatment can be undertaken that eliminates the bacillus before the immune conditions deteriorate and the risk of TB reactivation increases.

Currently, there are three commercial diagnostic tests for LTBI: the Tuberculin Skin Test (TST), which for decades was the only method for detecting TB infection, and two techniques introduced relatively recently that are based on the detection of interferon-gamma (IFN-G) released from sensitized lymphocytes against specific antigens of *M. tuberculosis:* Quantiferon^®^-TB Gold in-Tube (QFT) and T-SPOT^®^.TB (T-SPOT). These are all indirect methods of measuring infection by *M. tuberculosis*, as they detect the existence of an immune response against the bacillus as a surrogate marker of infection.^34^

The TST carried out using the Mantoux technique, measures the cell-mediated immune response against a standardized dose of purified protein derivative of tuberculin (PPD) injected into the dermis of the forearm of the patient. The existence of a palpable induration reaction after 48–72 hours, larger than 5, 10 or 15 mm, depending on the immune status of the patient, BCG vaccination and the epidemiological TB context, is suggestive of infection by *M. tuberculosis.*^35^,^36^ In general, an induration ≥10mm is considered to be indicative of LTBI in patients with HM.^37^,^38^ The major drawback of the TST is that most of the antigens present in PPD are not specific to *M. tuberculosis*, so that the specificity of the test is limited, especially for patients vaccinated with BCG (one of the most used vaccines in the world) or infected by non-tuberculous mycobacteria. In addition, there are also operational shortcomings: a second visit is necessary to assess the test 48–72 hours after the injection; measurement of the induration is subject to interobserver and interobserver variability; while the privacy of the result is on occasions difficult, because of the visible inflammatory reaction produced on the forearm. Nevertheless, it has the advantage of its low cost and the extensive experience gained over decades of use.

The Interferon-Gamma Release Assays (IGRAs) measure in vitro the release of IFN-G produced by T lymphocytes against the stimulation of highly specific (but not totally specific) antigens to *M. tuberculosis.* In order to carry out these tests, it is essential to obtain a blood sample from the patient. For T-SPOT, the process is more protracted because prior separation of the lymphocytes of the patient is required, whereas, for QFT, only a simple ELISA is needed.^39^ The antigens used with T-SPOT are ESAT-6 and CFP-10, while for QFT TB7.7 is also incorporated. These antigens are not present in the BCG strains used for vaccination, so that there are no false positive results in BCG vaccinated patients. Nevertheless, they are present (and can, therefore, cause false positive results) in some species of non-tuberculous mycobacteria, such as *M. Kansasii, M. marinum* and *M. szulgai*.

As mentioned above, all the current diagnostic tests for LTBI are based on the detection of an immune response by sensitized T lymphocytes that have been previously exposed to *M. tuberculosis*. Detection of their response against specific antigens of *M. tuberculosis* is indicative of TB infection. Therefore, the result obtained is only an indirect measurement of infection that can never be confirmed for LTBI. There is no available “gold standard” to evaluate the accuracy of these tests.

Overall, bearing in mind these limitations, IGRAs have shown sensitivity at least comparable to that of the TST for the diagnosis of LTBI, as well as greater specificity.^40^–^42^ Moreover, numerous studies have already demonstrated the predictive value of IGRAs regarding the risk of progressing to the disease, which is after all the final aim of tests for detecting LTBI.^43^–^45^ Taking into account the limitations for both TST and IGRAs, it has been suggested that a combined TST-IGRA testing is probably the best way for LTBI diagnosis in order to gain sensitivity in immunosuppressed patients.^38^,^46^

### Diagnosis of LTBI in patients with HM

The sensitivity of the tests for detecting LTBI in patients with underlying immunodeficiency is lower than that in patients with conserved immunity.^47^ But in contrast to patients with immunosuppression due to HIV, immune-mediated inflammatory disorders or pharmaceutical drugs, the number of studies that have evaluated the value of IGRAs in patients with HM is much more limited. Piana et al compared TST and T-SPOT in 138 Italian onco-hematological patients who had been in contact with a smear-positive TB patient. Of these patients, 17% were positive to the TST as against 44% who were positive to the T-SPOT test. Interestingly, a direct relationship was found between a positive T-SPOT result and the degree of exposure to the index case of TB, which suggests that the T-SPOT test has greater sensitivity than the TST in the diagnosis of LTBI in onco-hematological patients.^48^ Richeldi et al found a similar result in 95 Italian patients with HM, who simultaneously undertook the TST, QFT and T-SPOT tests. The percentage of positive results for IGRAs was significantly higher than that for the TST (26% for QFT, 18% for T-SPOT compared to 10% for the TST). However, this study did not analyse the correlation between the result of the tests and the degree of exposure (i.e. making it difficult to distinguish betweent rue positive result and a false positive one). The authors themselves recommend caution when interpreting the results, as it cannot be definitely stated that IGRAs have greater sensitivity than the TST for detecting LTBI in this group of patients.^49^ A recent study by Moon SM et al compared the TST and QFT in 244 South Korean patients who were candidates for hematopoietic stem cell transplantation. In contrast to the previous studies carried out with Italian patients, most of the patients had been vaccinated with BCG. The percentage of positive results was not significantly different (10% for the TST with a cut-off of 10mm against 16% for the QFT).^50^

### Indeterminate results

In addition to evaluating the production of IFN-G by T-lymphocytes in response to *M. tuberculosis* antigens*,* IGRAs also evaluate the production of IFN-G against a mitogen (Phytohemaglutinin, positive control) and a test tube without any antigen (null, negative control). If the patient has an immune response disorder, either due to lack of productions of IFN-G against the mitogen or due to an overproduction against the null control tube, an indeterminate result is produced, which means that the result obtained cannot be evaluated. Immunosuppressed patients present a higher prevalence of indeterminate results both with the T-SPOT test and QFT, normally because of a lack of responses to the positive control with mitogen. In a recent report of 18 children with cancer or primary immunodeficiencies who had been exposed to an adult with smear-positive TB, the T-SPOT test provided fewer indeterminate results than the QFT.^51^ In another study undertaken with 34 South African children with different types of cancer (21 with HM), indeterminate results were found in 4 patients (11%) with the T-SPOT technique, as against 15% when using the QFT. It should be stressed that, because of various problems, the T-SPOT technique could not be carried out with a further 7 children (21%) in this same study. Indeterminate results were more frequent in children with HM than in those affected by other types of malignancy.^52^

## Treatment of Latent Tuberculosis Infection

As mentioned earlier, patients with HM have a higher risk of TB reactivation than the general population. However, this does not necessarily mean that all patients with LTBI and HM should receive treatment for LTBI because, despite this higher risk, most patients with LTBI will never develop TB disease, even without preventive treatment. Treatment of LTBI may not be appropriate in certain situations such as a short life expectancy (which is the case for a relatively important percentage of patients with HM); when medical drug toxicity could be high, as in patients with underlying liver disease;^53^ or in patients for whom adherence to treatment could be suboptimal.^54^ decision should be made in an individualized and consensual way with each patient regarding whether or not to take treatment for LTBI. In our experience, the toxicity caused by the treatment of LTBI in patients with HM is no different to that for the population with other indications for treatment of LTBI.^55^

### Treatment regimens

There are several preventive treatment regimens with recognized efficacy in different risk situations for developing TB^46^,^56^,^57^ (Table 2). However, none of these has evaluated the efficacy of treatment specifically among patients with HM. Therefore, the recommendations for the treatment of LTBI in onco-hematological patients are based on studies carried out among patients in other risk situations for developing the disease.^58^–^61^ Prior to starting treatment of LTBI, it is necessary to exclude active TB. If there is an active TB, and a preventive treatment is started with only one drug, the patient is at high risk of generating a TB that is resistant to that drug.

The treatment of LTBI that has been most studied and has a proven efficacy is that with isoniazid (H). This treatment regimen should last between 6 and 9 months, while having the prolonged treatment the greatest preventive efficacy. The 9 months regimen reaches a protective efficacy against TB reactivation of 90% and is probably the best duration for LTBI treatment with isoniazid.^37^,^62^,^63^ An alternative is the use of rifampicin (R) for 4 months, which is especially recommended in cases of resistance to H.^64^,^65^ There is a third regimen of similar efficacy consisting of a combination of HR for 3 months. The shorter length of the treatment makes adherence easier.^66^ In a meta-analysis performed by Ena J et al in 2005, the development of active TB was found to be equivalent with short-course regimen with HR as compared to standard therapy with isoniazid (pooled risk difference: 0%; 95% CI: −1% to 2%).^67^ Finally, there is another shorter (3 months) effective alternative regimen with a single weekly dose of H and rifapentine (a rifamycin derivative still not widely used in Europe).^68^

## Active Tuberculosis

Infections are one of the most common complications of HM and are one of the main causes of mortality. Although bacteria are usually the aetiological agents,^19^,^69^ together with fungi and viruses to a lesser extent, the aetiology of other germs should always be considered. This applies to disease caused by *M. tuberculosis,* especially in the non-neutropenic phases and in countries with a high prevalence of TB.^20^,^25^,^26^,^70^–^72^

### Clinical aspects

TB patients with different types of cancer, including those with HM, often present clinical characteristics that are distinct to those of patients without any other underlying disease. The extra pulmonary form of TB is more frequent in these patients^20^,^25^ and in bone marrow transplant recipients.^73^ In addition mortality is higher, particularly in the extra pulmonary forms.^32^

### Diagnosis

The diagnostic gold-standard for active TB relies on the detection of *M. tuberculosis* by culture or molecular methods.^74^ Microscopy detection of acid-fast bacilli is a cheap and simple method for diagnosing TB, however, this method has limited sensitivity of 50% or less in sputum samples or even less in extra-pulmonary locations of TB.^75^ In addition, the presence of acid-fast bacilli is not always indicative of *M. tuberculosis* as other mycobacteria species have also this staining property. Nucleic acid amplification tests are increasingly used for rapid diagnosis of TB.^76^ One of these (Gen Xpert MTB/RIF®) is endorsed by the World Health Organization due to its simplicity of use, high sensitivity and because it also provides rapid diagnosis of TB. Additionally, this method, detects an eventual resistance to rifampicin in less than two hours after obtaining the sample.^77^–^79^ The diagnosis of non-pulmonary TB is often challenging, because it is not easy to obtain microbiological confirmation in such cases. It is not uncommon to recommend starting an empirical specific anti-tuberculosis treatment. Detection in urine of lipoarabinomannan (LAM), a constituent of the cell wall of *M. tuberculosis,* has shown its utility for TB diagnosis in HIV-infected patients. However, this method has not been validated in patients with HM.^80^ The suspected diagnosis of lymph node TB is especially difficult, because its presentation is similar to that of lymphoma. The presence of radiological hypodensity in the centre of the node is suggestive of necrosis and tuberculous aetiology, but by no means is it diagnostic.^81^ In cases where microbiological confirmation is not possible, there are various clinical and radiological characteristics that are suggestive of tuberculous aetiology.^82^–^84^ Nevertheless, for a definitive diagnosis, needle aspiration or needle biopsy is always required to obtain samples and to culture them in specific mycobacteria media.

As with the TST, the sensitivity of IGRAs for the diagnosis of TB is low in patients with immunosuppression. In a study in South Korea of patients with smear negative pulmonary TB and associated immunosuppression, the diagnostic sensitivity of T-SPOT and QFT among patients with a tumoral disease was 69% and 58%, respectively. These percentages were higher (although not reaching statistical significance) to the 37% obtained with the TST. In this same study, the authors conclude that neither the IGRAs nor the TST have value as a single test to rule out active TB in immunocompromised patients.^85^

## Treatment of Active Tuberculosis

The treatment of TB is based on two main principles: 1) the combination of at least 3–4 drugs, which avoids the emergence of bacterial resistance and 2) the need for prolonged treatments (of at least 6 months) to prevent relapses.^86^

The standard treatment regimen for previously untreated TB is the administration for two months of isoniazid, rifampicin, pyrazinamide and ethambutol (2HRZE) (intensive phase of treatment) followed by a continuation phase of 4 months with isoniazid and rifampicin (4HR).^87^,^88^ Ideally, the administration of the dose should be in a combined formulation (there are commercially available combinations of the 4 drugs in single presentations), in daily doses (although there are valid treatment regimens with doses taken two or three times a week), and should include measures that promotes adherence for the patient. Cure rates for sensitive strains with this regimen are higher than 98%.^86^,^89^ Rifampin is a potent inducer of both cytochrome P-450 oxidative enzymes and the P-glycoprotein transport system and may, therefore, decrease the level or effect of some chemotherapeutic agents prescribed in HM patients. Interactions of rifampin with bortezomib, doxorubicin, vincristine, vinblastine and bendamustine may be clinically significative.^90^,^91^

In certain circumstances, the standard regimen of 2HRZE+4HR cannot be used. The following are some of the most common causes:

### 1· Resistance or toxicity to any of the anti-tuberculosis drugs^88^,^92^

If isoniazid cannot be used, one of the following regimens is recommended: 6–9RZE or 2RZE+10RE.

In the situations where the rifampin cannot be used, the length of treatment should be extended up to 18 months. In addition, during the intensive phase, pyrazinamide should be included, as well as an injectable drug (preferably streptomycin, although amikacin; kanamycin and capreomycin also have a good anti-tubercular activity), or a third-generation quinolone (moxifloxacin) when there is a high bacillary burden.

If ethambutol cannot be used: 2HRZ+4HR.

If pyrazinamide cannot be used: 2HRE+7HR.

When neither isoniazid nor rifampicin can be used, combined resistance to these two drugs is known as Multidrug-resistant TB (MDR-TB) and is particularly challenging situation, as these two drugs have the highest activity against *M. tuberculosis*. In such scenarios, treatment should be individualized taking into account sensitivity to the other available drugs, and to the previous TB treatments. The length of the treatment should be at least 18–24 months and must be supervised by doctors with relevant experience. The treatment should include an injectable drug (streptomycin, kanamycin, amikacin or capreomycin) and a quinolone.^93^,^94^ The strains of MDR-TB that also present additional resistance to any quinolone and any of the three injectable drugs are known as extensively drug resistant TB (XDR-TB). In these circumstances, treatment is even more complicated and often leads to a poor prognosis.^95^–^97^

### 2· Slow microbiological response

The persistence of positive sputum cultures after two months of treatment has been related to an unacceptably high relapse rate. Therefore, it is recommended that the length of treatment be extended to a total of 9 months (2HRZE+7HR) in patients with drug-susceptible strains who have cavitation on the initial or follow-up chest radiograph and are culture-positive at the second month of treatment.^98^

### 3· Other scenarios

In patients with HIV co-infection and absence of antiretroviral treatment, in cases of silicosis, or in cases of bone and joint or meningeal TB, the prolongation of treatment beyond 6 months has been suggested with the aim of preventing relapses.^99^,^100^

For patients with TB and HM, there is no evidence that the regimen or length of treatment should differ from the standard regimen.

## Figures and Tables

**Table 1:** Studies that have evaluated the risk of tuberculosis in patients with haematological malignancies

Reference	Setting	RR	Comments
**Libshitz HI**^29^	USA	9.0	Cancer patients (including HM)
**de la Cámara**^30^	Spain	2.95	Allogenic Stem Cell Transplantation
**Kamboj M**^27^	USA	40	Highest rate among Stem Cell Transplant Recipients
**Mishra P**^31^	India	23	Patients with acute leukemia. Highest rate in AML
**Stefan DC**^26^	South Africa	22	Children with LLA
**Chen CY**^32^	Taiwan	2.05	
**Wu CY**^21^	Taiwan	3.22*	Non-Hodgkin Lymphomas and Leukaemias

RR: Relative Risk compared to the general population. AML: Acute Myeloid Leukemia. HM: Haematological Malignancies.

* Hazard Ratio

**Table 2:** Treatments recommended for Latent Tuberculosis Infection

DRUG REGIMEN	LENGTH OF TREATMENT	ADULT DOSE	SIDE EFFECTS COMMENTS
**INH**	6–9 months	300mg/day	Hepatotoxicity Neurotoxicity
**RIF**	4–6 months	600 mg/day	Hepatotoxicity Thrombopenia Interaction with other drugs
**INH+RIF**	3 months	Idem as above	Not recommended in HIV
**INH+RPT**	3 months	900mg+900mg per week	DOT
**PZA+RIF**			No longer used because of toxicity

INH: isoniazid; RIF: rifampicin; RPT: rifapentine; PZA: pyrazinamide; DOT: Directly Observed Therapy.
